# Doege-Potter Syndrome; A Case of Solitary Fibrous Pleura Tumor Associated with Severe Hypoglycemia: A Case Report in Internal Medicine

**DOI:** 10.2174/1871530323666230623112047

**Published:** 2023-09-15

**Authors:** Viviana Castaldo, Daniela Domenici, Mauro Valentino Biscosi, Paolo Ubiali, Cesare Miranda, Giorgio Zanette, Cinzia Mazzon, Maurizio Tonizzo

**Affiliations:** 1 Department of Internal Medicine, Hospital Santa Maria degli Angeli, Pordenone, Italy;; 2 Department of Endocrinology and Metabolic Diseases, Hospital Santa Maria degli Angeli, Pordenone, Italy;; 3 Department of Radiology, Hospital Santa Maria degli Angeli, Pordenone, Italy;; 4 Department of General Surgery, Hospital Santa Maria degli Angeli, Pordenone, Italy

**Keywords:** Hypoglycemia, doege-potter syndrome, non-islet cell tumor hypoglycemia, solitary fibrous tumor, IGF-2, STAT6

## Abstract

**Background:**

Doege-Potter syndrome is a rare paraneoplastic entity that is often diagnosed incidentally during the work-up of hypoglycemia of unclear etiology. It is characterized by a non-islet cell tumor hypoglycemia mostly associated with solitary fibrous tumors. These uncommon tumors have been reported in <5% of solitary fibrous tumors. Although not unique in its kind, this case is extremely important as this syndrome often conceals unrecognized tumors that can be surgically resolved.

**Case Presentation:**

We present the case of a 59-year-old non-diabetic man with a 2-month history of severe and recurrent fasting hypoglycaemia presenting with severe dyspnea and sweating. Further work-up revealed low insulin, C-peptide, and IGF-1 levels and a large right in-trathoracic solitary fibrous tumor. Unfortunately, bioassays for IGF-2 were unavailable at our hos-pital. Nevertheless, as hypoglycemia completely resolved after resection of the mass, Doege-Potter syndrome was highly suspected.

**Conclusion:**

Doege-Potter syndrome is a complication of rare tumors. If hy-poglycemia is unexplained, this syndrome should always be suspected, and the presence of un-known masses should be investigated.

## 1 INTRODUCTION

Hypoglycemia is a medical emergency commonly caused by drugs (*e.g*., insulin, sulfonylureas), alcohol, and critical illness (Table **1**). Occasionally, it can manifest endogenous hyperinsulinism (*e.g*., insulinoma). In less than 5% of cases, non-islet cell neoplasms, such as solitary fibrous tumors (SFT), can cause hypoglycemia. This condition is called Doege-Potter syndrome (DPS) [1]. DPS is a paraneoplastic disorder with recurrent non-islet cell tumor hypoglycemia (NICTH) due to excessive secretion of precursor “big” insulin-like growth factor II (IGF-2) from the SFT [2]. This syndrome was first described in 1930, and to date, only a few cases of DPS have been reported worldwide [3-5].

SFTs are rare neoplasms that arise from mesenchymal cells, accounting for less than 2% of soft tissue tumors. In more than 80% of the cases, SFTs are characterized as slow-growing, indolent neoplasms with low malignant potential [6].

Most SFTs are primarily located in the pleura (referred to as SFTP) and typically present as incidental findings. However, in some cases, SFTPs can be associated with respiratory symptoms due to the compression of lung tissue by the pleural mass. According to Han *et al*., the most common cause of Doege-Potter syndrome is SFTP involving the right hemothorax [7].

Recurrent fusion transcripts caused by inversions at chromosome 12q13 have been associated with SFTs. More specifically, the NAB2-STAT6 gene fusion, resulting in a chimeric protein (STAT6), has been identified as the hallmark of the SFT [8].

Here, we report a case of presumptive Doege–Potter syndrome secondary to SFTP in a 59-year-old male with complete resolution of hypoglycemia after resection of the pleural tumor.

## 2 CASE PRESENTATION

A 59-year-old male was referred to Santa Maria degli Angeli Hospital, Pordenone, Italy, with a complaint of recurrent and documented fasting hypoglycemia that had been occurring for the past two months. He reported experiencing excessive sweating, tremors, and weakness during these episodes. Finger stick tests were used to confirm the hypoglycemia, with blood glucose levels ranging from 20 to 50 mg/dL.

The symptoms generally resolved after breakfast, and the postprandial glucose level reached the peak of about 70 mg/dL, which was determined with a fingerstick test. His medical history included hypertension and benign prostatic hyperplasia. He chronically consumed nebivolol, hydrochlorothiazide, and tamsulosin. He did not suffer from diabetes mellitus and denied the intake of any glucose-lowering or herbal medication. He was not a smoker and did not consume alcohol or illicit drugs. His family history was unremarkable. His recent history was notable for frequent sudden onset of severe dyspnea and sweating, with no accompanying chest pain or weight loss.

During the lung examination, severely diminished breath sounds were detected in all areas of the right lung, along with dullness upon percussion. No superficial lymph node enlargement was observed. Both cardiovascular and abdominal examinations yielded unremarkable findings.

A biochemical work-up for hypoglycaemia revealed non-insulin-mediated hypoglycemia with a blood glucose level of 25 mg/dL (normal range: 70-99 mg/dL). Furthermore, there were concomitant low serum insulin levels (1 microIU/mL, normal range: 6-27 microIU/mL), low serum C-peptide levels (<0.5 microg/L, normal range: 0.5-4.0 microg/L), and low serum IGF-1 levels for sex and age (60 mcg/L, below the 1st percentile). The pituitary function was found to be normal, and cortisol deficiency was ruled out. Additionally, insulin antibodies tested negative. The renal and hepatic functions were within normal limits. A complete blood count and metabolic panel excluded anemia and electrolyte imbalances (Table **2**).

**Fig. (1):**
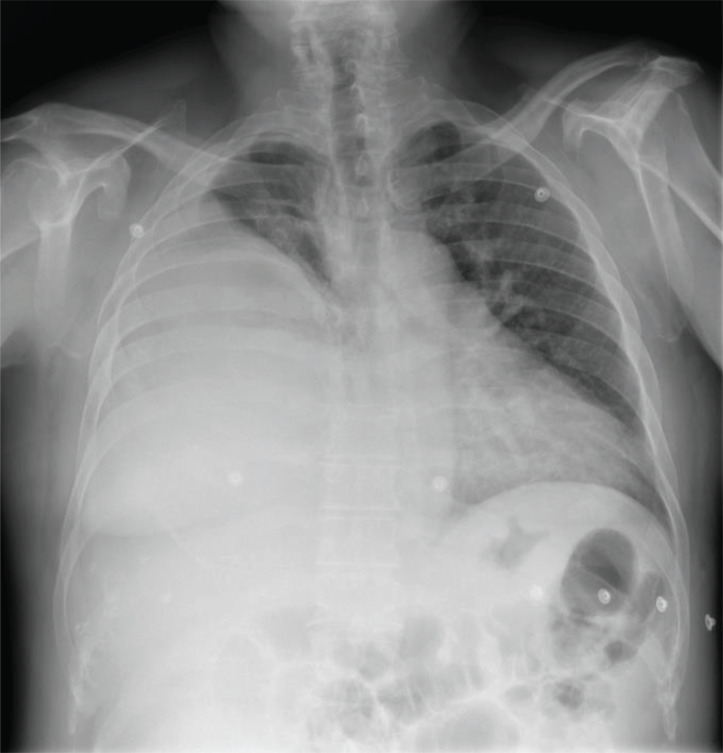
Chest X-ray showing a well-demarcated homogeneous space-occupying lesion. Trachea is central. The right principal bronchus is not well defined. Cardiomediastinal shadow is lightly pushed on left side.

Chest X-ray showed a sub-total white right hemithorax with loss of volume lung (lung consolidation; pleural mass; chest wall mass) (Fig. **1**).

Microbiological tests, including *Legionella pneumophila* and *Streptococcus pneumonia* urine antigens, multiplex real-time polymerase chain reaction (PCR) for respiratory virus detection and blood cultures, all yielded negative results.

**Fig. (2):**
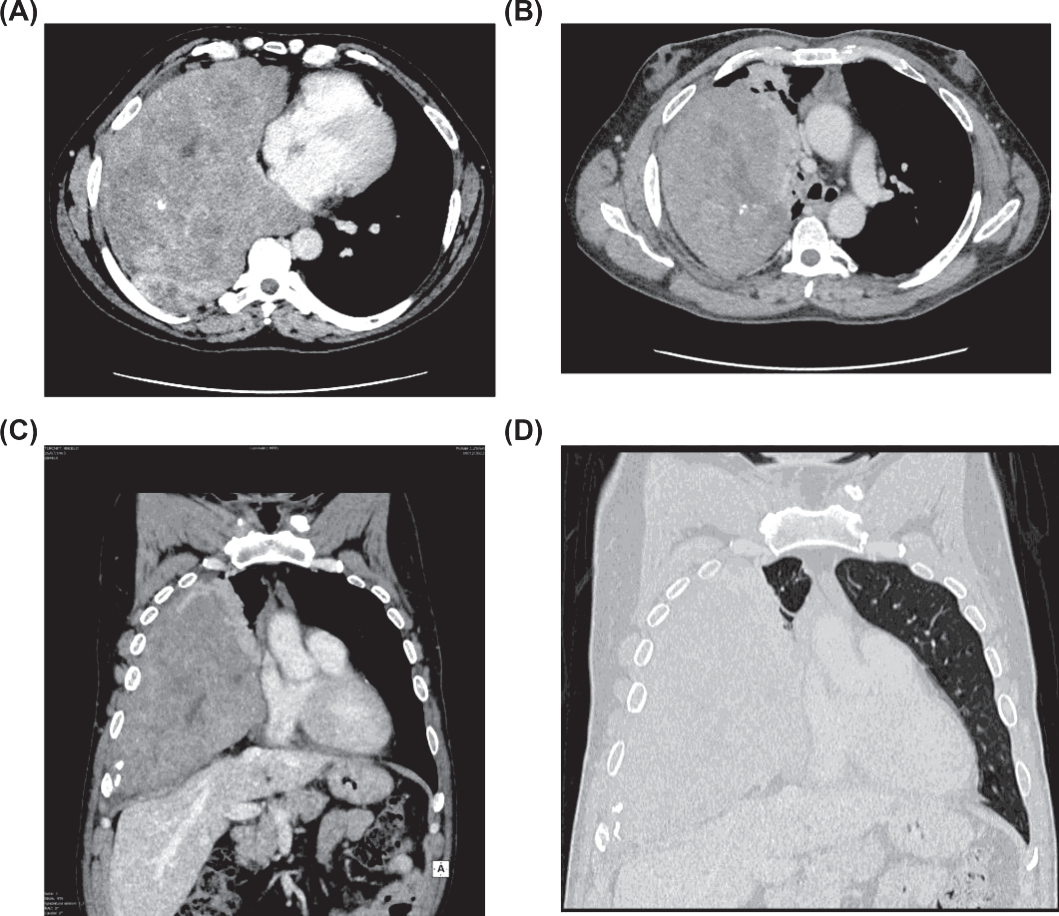
CT (computed tomography) and MPR (multiplanar reconstruction) images. (**A, B**) Large heterogeneously enhanced mass with superior vena cava and right atrium compression. (**C, D**) MPR: note parenchymal lung compression not involved right apical segment.

To further characterize the condition, a chest/abdomen CT scan was performed. The CT images revealed a large heterogeneous enhancing mass measuring 22.5×16.5×22.5 cm in the right lower hemithorax. The mass did not involve the rib cage but exerted pressure on the adjacent lung and cardiomediastinal structures, including the superior vena cava, right atrium, and right pulmonary vein. A large pleural fibroma was hypothesized based on these findings (Fig. **2**). CT scan did not show any adenopathy, other mass lesions, or bone metastasis.

As a result, a diagnosis of Doege-Potter syndrome was strongly suspected. However, due to the unavailability of an IGF-2 bioassay test at our hospital, we were unable to calculate the IGF-2:IGF-1 ratio, which serves as a confirmatory diagnostic test for Doege-Potter syndrome.

**Fig. (3):**
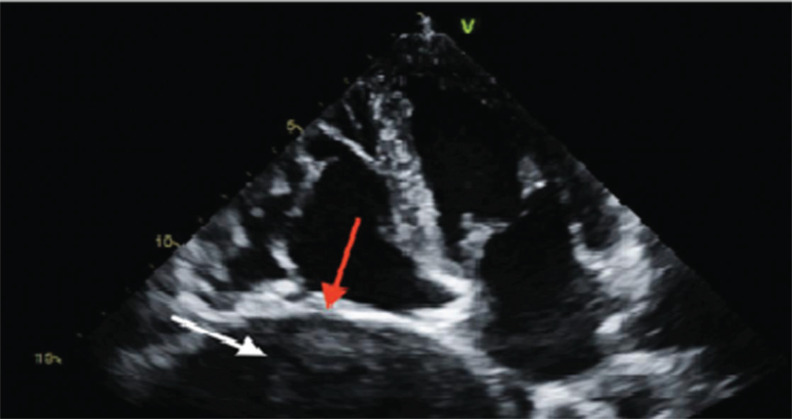
Transthoracic echocardiogram showing lung mass.

Transthoracic echocardiography was also performed to rule out a cardiogenic cause of the dyspnea. A large mass compressing the right atrial cavity was revealed (Fig. **3**).

The bronchoscopic evaluation revealed a friable mass. However, cytology did not detect any cancerous cells, and the biopsy yielded inconclusive results. Due to the high suspicion index, another biopsy was performed under the CT-scan guide. Microbiological causes investigated by means of Gram stain, Acid-fast bacteria (AFB) stain, AFB culture, and fungal culture all came back negative.

The histological evaluation showed a tumor consisting of simple spindle-shaped cells with no apparent nuclear pleomorphism. There was no evidence of necrosis. Immunohistochemical analysis revealed the following findings: Bcl-2 (+), STAT6 (+), CD34 (+), S100 (-), CK7 (−), WT1 (−), D2-40 (−), calretinin (−), MDM2 (−). These findings, along with the presence of non-islet cell tumor hypoglycemia (NICTH) in the context of SFTP, supported the diagnosis of Doege-Potter syndrome.

**Fig. (4):**
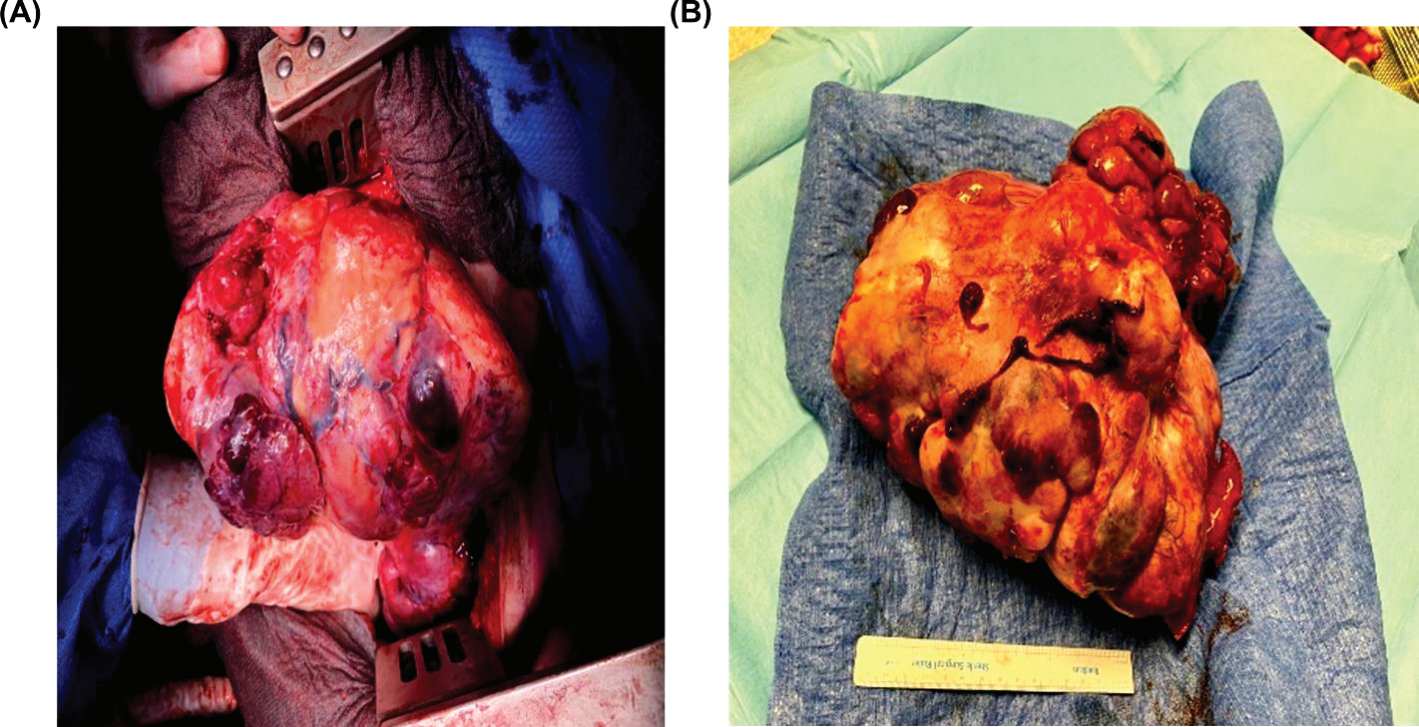
Surgical resection of the tumor (**A**) and gross appearance of the mass (>20 cm) post-resection (**B**).

A continuous infusion of 10% glucose was promptly administered, and the patient was advised to have frequent meals. However, episodes of hypoglycemia continued to occur. Due to the recurrence of symptoms, the medical team proposed a surgical approach. Consequently, the patient underwent a right posterolateral open thoracotomy for mass resection. The tumor, originating from the anterior mediastinum, was located within the right lung. A portion of the lower lobe of the right lung was resected. The encapsulated mass measured 25.3×19×17 cm and weighed 3720 g (Fig. **4**).

Histological examination of the surgical sample confirmed the CT-guided biopsy findings, consistent with SFT. Patient’s age >55 years, tumor size >15 cm, Ki-67 proliferation index of 10-12%, mitotic activity >4/10 high-power fields (HPF) and necrosis rate <10% classified this SFT as a tumor at high risk of recurrence/metastasis (according to WHO classification).

**Fig. (5):**
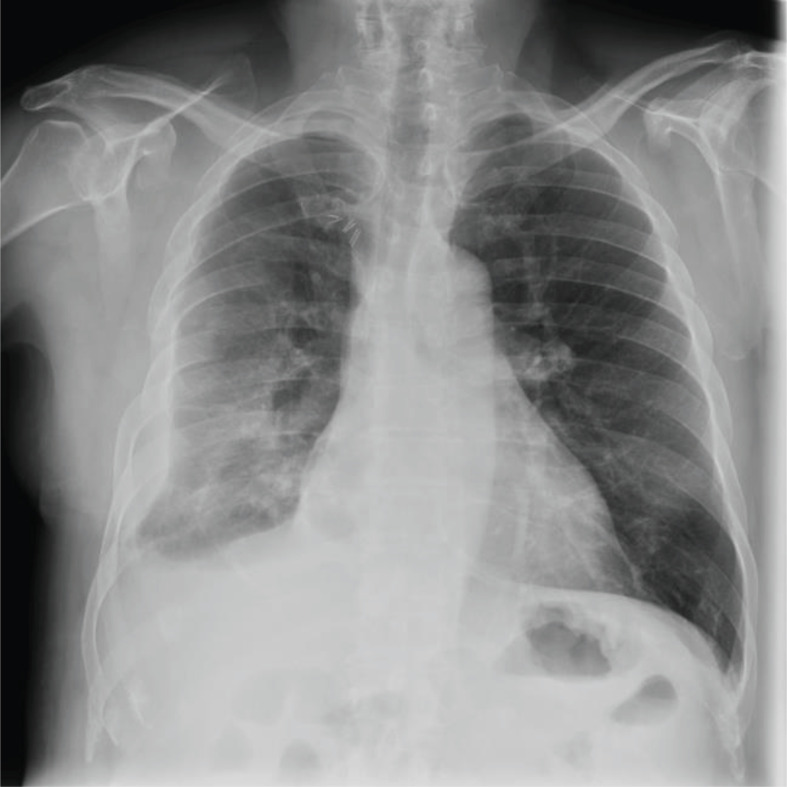
Chest X-ray after resection (1 month).

A multidisciplinary team did not consider any adjuvant treatment necessary. Post-operative serum glucose levels normalized, and the patient was discharged after a few days without re-presenting hypoglycemic episodes. Dyspnea completely resolved as well. Two weeks after discharge, the chest radiograph showed pulmonary re-expansion in the right hemithorax (Fig. **5**).

## 3 RESULTS AND DISCUSSION

Hypoglycemia is a medical emergency that, in less than 5% of cases, can be caused by a non-islet cell tumor (NICTH), most commonly associated with solitary fibrous tumors (SFT).

Doege-Potter syndrome (DPS) is a rare paraneoplastic entity characterized by NICTH due to excessive secretion of precursor “big” insulin-like growth factor II (IGF-2) from the SFT. This prohormone suppresses growth hormone and leads to decreased synthesis of IGF-binding proteins, resulting in elevated levels of unbound and active IGF-2 [9]. The binding of active complexes or unbound IGF-2 to insulin and IGF receptors in the liver and peripheral tissues causes a decrease in glucose release into the bloodstream and an increase in peripheral glucose metabolism, leading to hypoglycemia [10].

We present a case of suspected Doege-Potter syndrome associated with a solitary fibrous tumor of the pleura.

During the diagnostic process, various causes of hypoglycemia were ruled out. The patient exhibited very low levels of insulin and C-peptide, prompting an investigation into common causes of non-insulin mediated hypoglycemia, such as critical illness, renal or liver failure, and hormonal deficiencies (*i.e*., cortisol, GH). A history of anti-diabetic drugs or alcohol use was also excluded. The biochemical work-up revealed a deficiency in growth hormone (GH) and insulin-like growth factor-1 (IGF-1) for the patient's sex and age.

Unfortunately, IGF-2 determination could not be obtained as the specific immunoassay kit for this protein was unavailable at our hospital.

Nonetheless, the results of all the available tests were consistent with a diagnosis of DPS, including recurrent, refractory fasting and non-insulin-mediated hypoglycemia, radiological and histopathological detection of a solitary fibrous tumor, and the complete resolution of hypoglycemia following surgical resection of SFT.

SFTs are uncommon tumors with a mesenchymal origin, first described by Wagner in 1870. They often originate from the pleura, and approximately 12–13% of the cases are malignant neoplasms [11].

DPS is more commonly observed in individuals between the sixth and eighth decade of life, and the incidence of SFTP with DPS is similar between genders [12].

Tumors causing NICTH have been found to express high levels of IGF-2 mRNA, attributed mainly to mutations or loss of imprinting in tumor suppression genes, resulting in overproduction of pro-IGF-2 [13].

SFTs can remain completely asymptomatic or present with recurrent hypoglycemia. Diagnosis requires imaging and histopathological examination. Intrathoracic SFTs typically appear as large, well-circumscribed, lobular, solitary nodules or masses in the lung periphery, often in close proximity to the pleural surface [12]. Microscopic examination reveals a bland spindle-cell proliferation arranged in fascicles, with areas of hypo- and hyper-cellularity separated by thick and thin collagen bundles. Mitotic activity is usually low (<3/10 high-power fields, HPF), cytologic atypia is minimal, and necrosis is infrequent [14, 15].

In our case, a Ki-67 proliferation index of 10-12% and a mitotic activity >4/10 high-power fields (HPF), together with a tumor size >15 cm and a patient’s age >55 years, suggested a high risk of recurrence/metastasis, according to WHO criteria [7].

## CONCLUSION

The most characteristic immunohistochemical finding in DPS is the CD34 expression; however, approximately 5% of STFs may be negative for this marker. The NAB2-STAT6 gene fusion, resulting in a chimeric protein (STAT6), has been identified as a consistent finding in SFTs. Nuclear expression of the carboxy-terminal part of STAT6 serves as a highly sensitive and specific immunohistochemical marker for SFT, aiding in the differentiation of this tumor type from histologic mimics [16]. In our case, immunohistochemistry for both CD34 and STAT6 was positive, further supporting the diagnosis of SFT.

**Fig. (6):**
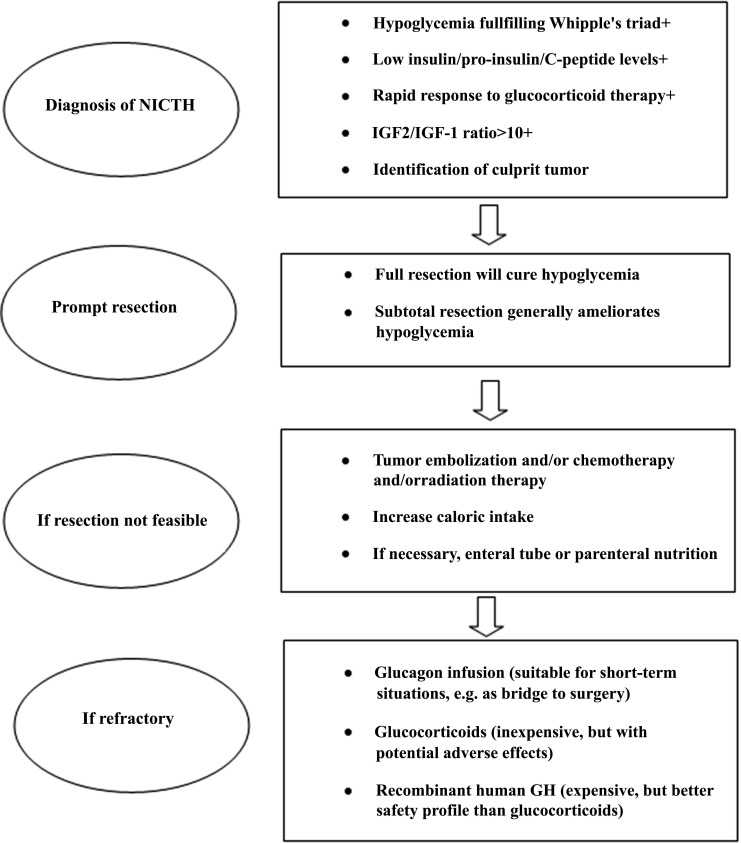
Diagnostic and therapeutic algorithm for DPS. Adapted from Bodnar TW *et al*., J Clin Endocrinol Metab. 2014 [18].

Therapeutic strategies for DPS (Fig. **6**, Table **3**) can be categorized into approaches that directly reduce the tumour burden (such as surgery and chemotherapy) and treatments aimed at mitigating hypoglycemia without treating the underlying tumour [17-19].

Complete surgical resection is the definitive curative approach for both benign and malignant SFTs. Resectability is a crucial prognostic factor, and the overall long-term cure rate after complete resection in all patients ranges from 80% to 92% [17]. Furthermore, the clinical management of hypoglycemia includes oral or intravenous glucose therapy (as was administered to our patient), corticosteroids and somatostatin analogs.

In our case, surgical resection of the thoracic mass resulted in a complete resolution of hypoglycemia. Although not unique in its kind, this case is extremely important for the scientific community as it presents a very rare syndrome that internists should always consider. This syndrome often conceals unrecognized tumors that can be surgically resolved.

## Figures and Tables

**Table 1:** Main causes of hypoglycemia.

**Insulin-mediated**	**Non-insulin Mediated**
Insulinoma Insulin autoimmune hypoglycemia	Hepatic, renal or cardiac failure
Hypoglycemia factitia	Critical illness (e.g. sepsis, lactic acidosis)
Post-bariatric surgery hypoglycemia	Malnutrition
Dumping syndrome	Cortisol, growth hormone or glucagon deficiency
Non-insulinoma pancreatogenous hypoglycemia (nesidioblastosis)	Non-islet cell tumor hypoglycemia (NICTH); Alcohol
Diabetes mellitus type 2 Idiopathic	Anti-diabetic drugs/other drugs

**Table 2:** Results and reference ranges of the laboratory tests performed.

**zTest**	**Values**	**Reference Range**
Glucose (mg/dL)	25	70-99
Insulin (microIU/mL)	1	6-27
C-peptide (microg/L)	<0.5	0.5-4.0
Insulin antibodies (U/mL)	0	0-10
IGF-1 (microg/L)	60	45-260
GH (ng/mL)	0.9	0.0-4.0
HbA1c (mmol/mol)	36	20-42
TSH (mIU/L)	1.5	0.20-3.75
Serum cortisol (microg/dL)	24	8-25
ACTH (ng/L)	39.4	10-60
FSH (U/L)	6.1	1.5-18
LH (U/L)	4.6	1.5-9
Total testosteron (microg/L)	4.2	1.8-7.5
WBC (10^3/IU)	4.11	3.99-10.0
HB (g/dL)	12.5	13.5-17.0
Creatinine (mg/dL)	0.69	0.7-1.2
AST (U/L)	17	<40
ALT (U/L)	25	<78
Bilirubin (mg/dL)	0.5	0.2-1
INR	1.13	0.95-1.2
Sodium (mmol/L)	142	135-145
Potassium (mmol/L)	3.5	3.5-5.0
Total cholesterol (mg/dL)	149	<200
PCR (mg/dL)	<0.3	0.0-0.5
PCT (microg/L)	0.04	0.02-0.05
LDH (U/L)	253	80-300
Chromogranin A (ng/mL)	74	0.0-108
NSE (ng/mL)	16	0.0-18
CEA (microg/L)	1	0.0-5
AFP (microg/L)	1.1	0.0-10
CA 19.9 (U/mL)	12.8	0.0-37
CA 15.3 (U/mL)	12.5	0.0-31

**Abbreviations**: GH: Growth Hormone; HbA1c: Glycated Hemoglobin; TSH: Thyroid Stimulating Hormone; ACTH: Adrenocorticotropic Hormone; FSH: Follicle Stimulating Hormone; LH: Luteinizing Hormone; WBC: White Blood Cells; HB: Hemoglobin; AST: Aspartate Aminotransferase; ALT: Alanine Transaminase; INR: International Normalized Ratio; PCR: C-reactive Protein; PCT: Procalcitonin; LDH: Lactate Dehydrogenase; NSE: Neuron-specific Enolase; CEA: Carcino-embryonic Antigen; AFP: Alpha-fetoprotein; CA: Cancer Antigen; IU: International Unit.

**Table 3:** Therapeutical strategies for DPS.

**Strategy**	**Advantages**	**Disadvantages**
Surgery	Surgery is generally sufficient to eradicate the tumor and resolve hypoglycemia	Invasive and risky; may not feasible (*e.g*. malignant tumors, with metastasis)
Chemotherapy	May be used to treat non resectable tumors	DPS related tumors are typically poorly responsive to systemic chemotherapy; chemotherapy regimens are not well studied; significant side effects associated with chemotherapy
Scheduled snacks	Non-invasive	Relies on patient adherence to schedule
Dextrose infusion	Prevents hypoglycemia	Requires long term venous access ; risky (*e.g*. infections)
Nocturnal or continuous enteral tube feeding	Prevents hypoglycemia	Long term use requires placement of gastrostomy tube
Corticosteroid administration	Non invasive; may normalize insuline-like growth factor levels; increases appetite	Multiple adverse effect from long term use
Continuous glucagon infusion	Effective to prevent hypoglycemia in some patients	May be pratically difficult; ;subcutaneus administration has less infectious risk than direct venous access

**Note:** Adapted from Schutt RC *et al*., Journal of Medical Case Reports. 2013 [19].

## LIST OF ABBREVIATIONS

CT: Computerized Tomography
DPS: Doege-Potter Syndrome
IGF: Insulin-like Growth Factor
NICTH: Non-islet Cell Tumor Hypoglycemia
SFT: Solitary Fibrous Tumor
SFTP: Solitary Fibrous Tumor of the Pleura
STAT6: Signal Transducer and Activator of Transcription 6
WHO: World Health Organization.

## STANDARDS OF REPORTING

CARE guidelines were followed in this study.
